# A Chinese Herbal Formula, Gengnianchun, Ameliorates *β*-Amyloid Peptide Toxicity in a* Caenorhabditis elegans* Model of Alzheimer's Disease

**DOI:** 10.1155/2017/7480980

**Published:** 2017-10-11

**Authors:** Fanhui Meng, Jun Li, Yanqiu Rao, Wenjun Wang, Yan Fu

**Affiliations:** ^1^Department of Gynecology, The First Hospital of Jilin University, Changchun, China; ^2^Department of Integrated Traditional Chinese Medicine and Western Medicine, Obstetrical and Gynecological Hospital, Fudan University, Shanghai, China

## Abstract

Alzheimer's disease (AD) is an age-related neurodegenerative disorder, and the few drugs that are currently available only treat the symptoms. Traditional medicine or phytotherapy has been shown to protect against AD. In our previous studies, Gengnianchun (GNC), a traditional Chinese medicine formula with a prolongevity effect, protected against A*β*-induced cytotoxicity in pheochromocytoma cells (PC-12 cells) and hippocampal cells. Here, we investigated the effects and possible mechanisms by which GNC protected against A*β* toxicity using transgenic* Caenorhabditis elegans* CL4176. Our results showed that GNC effectively delayed the A*β* toxicity-triggered body paralysis of CL4176 worms. GNC decreased A*β* by reducing A*β* mRNA levels. Moreover, GNC significantly reduced reactive oxygen species in the AD model worms compared with the controls. In addition, GNC upregulated the daf-16, sod-3, hsp-16.2 genes, and enhanced DAF-16 translocation from the cytoplasm to the nuclei under oxidative stress conditions. GNC treatment of* C. elegans* strains lacking DAF-16 did not affect the paralysis phenotype. Taken together, these findings suggest that GNC could protect against A*β*-induced toxicity via the DAF-16 pathway in* C. elegans*. Further studies are required to analyze its effectiveness in more complex animals.

## 1. Introduction

Alzheimer's disease (AD) is a chronic, progressive neurodegenerative disorder with a prevalence of 5% in people over 60, and the risk increases with age [1]. As the global population lives longer, AD has become a great burden to society, particularly countries in which ageing individuals represent a large proportion of the population [2]. Currently, a limited number of effective therapeutics for AD are available, and only a few drugs have been approved by the FDA as AD treatments, such as acetylcholinesterase (AChE) inhibitors and the N-methyl-D-aspartate receptor antagonist memantine [3, 4]. However, these drugs only alleviate symptoms for a short period, and their side effects are unfavourable. Hence, additional effective drug candidates must be identified to combat AD.

Traditional Chinese medicine and other natural herb-based therapies have been reported to be effective in preventing or protecting against AD for many years. For example, a recombinant buckwheat trypsin inhibitor (rBTI) was shown to protect against AD by promoting the activity of the autophagy-lysosomal degradation pathway [5].* Lycoris* compounds were shown to modulate inflammatory- and stress-related gene expression to alleviate *β*-amyloid induced toxicity in* C. elegans* [6]. Gengnianchun (GNC), a traditional Chinese herbal medicine formula, has been clinically used for more than 20 years in China. The GNC formula is composed of Radix Rehmanniae, Rhizoma Coptidis, Radix Paeoniae Alba, Rhizoma Anemarrhenae, Cistanche Salsa, Radix Morindae Officinalis, Poria, Epimedium Brevicornum, Cortex Phellodendri Amurensis, Fructus Lycii, Semen Cuscutae, and Carapax et plastrum Testudinis. It is currently used in the clinic to alleviate declining functions related to ageing. As shown in our previous studies, GNC enhanced the performance of ovariectomized rats in the Morris water maze test and elevated the activities of hippocampal acetylcholine (ACh), acetylcholinesterase (AChE), and choline acetyltransferase (ChAT) [7]. In addition, GNC upregulated brain-derived neurotrophic factor (BDNF) expression and improved learning and memory in ovariectomized rats [8]. GNC has recently been shown to attenuate A*β*_25–35_-induced cytotoxicity in pheochromocytoma cells (PC-12 cells) and hippocampal cells [9, 10]. Ageing and oxidative stress are known to have important roles in the progression of AD [11, 12]. GNC enhances the resistance to oxidative stress and improves lifespan* in vivo* [13]. Based on these results, GNC may be a promising therapeutic agent for the treatment of AD.

Although the aetiology of AD remains unclear, the most widely accepted hypothesis is the “amyloid hypothesis,” which suggests that A*β* neurotoxicity plays a central role in the pathogenesis of AD [14]. The toxic beta amyloid (1–42) peptide is derived from the abnormal processing of the amyloid precursor protein (APP), which forms extracellular senile plaques in the human brain and leads to downstream neurotoxic events, including neuronal dysfunction, stress responses, and inflammation [15].

Furthermore, oxidative stress is extensive in Alzheimer's disease brains. *β*-Amyloid accumulation is associated with markers of oxidative stress, including protein oxidation, lipid peroxidation, and oxidation of nucleic acids [16–18]. A*β* has been shown to induce oxidative stress in vitro and in vivo [19]. In vitro cells exposed to A*β* showed high levels of hydrogen peroxide and production of reactive oxygen intermediates [20, 21]. Previous in vivo studies have reported an increase in H_2_O_2_ levels and in the peroxidation of proteins and lipids in AD transgenic mouse models that express mutant amyloid precursor protein (APP) and presenilin-1 (PS-1) [22, 23]. In AD patients, A*β* accumulates in human brain mitochondria, resulting in a significant decline in mitochondrial function, which can result in increased generation of ROS [24, 25].

The transgenic* Caenorhabditis elegans* model has been used to evaluate the efficacy of anti-AD drugs since 1995 [26]. The transgenic* C. elegans* strain CL4176 expresses human A*β*_1–42_ in muscles under a temperature-inducible system, and the expression and subsequent aggregation of A*β* result in paralysis. In transgenic CL4176 worms, Ginkgo biloba extract EGb 761 [27], Liuwei Dihuang [28], cocoa peptide [29], and other compounds [5, 30] were shown to significantly reduce A*β* toxicity and ameliorate AD symptoms.

In the present study, we used transgenic* C. elegans* CL4176 to investigate whether GNC exhibits protective effects against A*β* toxicity in vivo and to elucidate some of the mechanisms involved in its protective effects.

## 2. Materials and Methods

### 2.1. Preparation of the GNC Formula

GNC formula is composed of 12 crude herbs. The preparation of the GNC formula has been reported in our previously published paper [13]. The composition of GNC followed traditional Chinese medicinal theory, and the composition is derived from our clinical experience. In this study, we used a mixture of the water extracts of 12 crude herbs. The water extracts were purchased from Tianjiang Pharmaceutical (Jiangyin, China). These products were manufactured with rigid quality control protocols following the rigid specifications of the Pharmacopeia of China. The water extracts in this study were produced in the same batch.

### 2.2. *C. elegans* Strains and Maintenance

The wild-type* C. elegans* strain N2 (Bristol), transgenic* C. elegans* strain CL4176 (smg-1ts [myo-3/A*β*1–42 long 3′-untranslated region (UTR)]), TJ356 (zIs356 [daf-16 p::daf-16a/b::GFP + rol-6(su1006)]), and* E. coli* OP50 were obtained from the Caenorhabditis Genetics Center (CGC) at the University of Minnesota (Minneapolis, MN, USA). CL4176 is a temperature-sensitive mutant; when the temperature is increased from 16°C (permissive temperature for the smg-1ts mutation) to 25°C (a nonpermissive temperature), the worms will express higher levels of the human A*β*_1–42_ peptide in muscles. CL4176 were maintained at 16°C on solid nematode growth medium (NGM) plates seeded with* E. coli* OP50, whereas wild-type worms N2 and TJ356 strain were maintained at 20°C.

### 2.3. Paralysis Assays

Synchronized CL4176 gravid adults were picked onto plates to lay eggs, and after 3-4 h, worms were removed, and eggs were maintained at 16°C on solid NGM plates that contained the vehicle control (H_2_O) and different concentrations of GNC (0.0394, 0.394, 1.97, 3.94, and 7.88 mg/mL). The nematodes were maintained at 16°C for 48 hours; then, transgene expression was induced by increasing the temperature from 16°C to 25°C. Each worm was gently touched with a platinum loop to confirm paralysis, and the nematode was scored as paralyzed if it only moved its head or did not show a full body wave. Paralyzed nematodes were counted at 1 hour intervals from 28 to 38 h after the temperature was increased to 25°C.

### 2.4. Western Blotting of A*β* Species

The A*β* species in the transgenic* C. elegans* strains were identified by immunoblotting using a Tris-Tricine gel and a standard Western blotting protocol [31]. Worms were harvested by washing with M9 buffer, centrifuged at 12,000 rpm for 15 minutes, and sonicated in RIPA lysis buffer (Pierce, Rockford, IL, USA) containing a protease inhibitor cocktail for 30 minutes at 4°C. The protein concentrations were quantified using a Bradford Protein Assay Kit (Beyotime, Shanghai, China). Before loading, samples were boiled with loading buffer at 100°C for 5 minutes. Equal amounts of protein were loaded in each lane of the Tricine-SDS-PAGE gel (consisting of a 10% “spacer gel” between a 4% stacking gel and 16% separating gel), and electrophoresis was started with an initial voltage of 30 V and maintained at this voltage until the samples have completely entered the stacking gel, and then the samples were run at 120 V for approximately 8 hours. The gel was transferred onto polyvinylidene fluoride (PVDF) membranes. PVDF membranes were blocked with 5% non-fat milk in Tris-buffered saline with Tween (TBST) (100 mM Tris, pH 7.5, 150 mM NaCl, 0.1% Tween-20) overnight at room temperature. Amyloid protein species were detected with a BAM-10 monoclonal antibody (1 : 1,000; Sigma) and a peroxidase-conjugated secondary anti-mouse IgG (1 : 5,000; Sigma). Actin was detected with an anti-actin antibody (1 : 1,000, Abcam) and was used as an internal control. The mean optical density was analyzed using ImageJ software (US National Institutes of Health, Bethesda, MD, US). Three independent experiments were conducted and consistent results were obtained from these independent experiments.

### 2.5. Measurement of Reactive Oxygen Species (ROS) Levels in* C. elegans*

Intracellular ROS levels in* C. elegans* CL4176 were measured using 2,7-dichlorofluorescein diacetate (H_2_DCF-DA), according to a previously reported method [6]. The procedures used for GNC administration were the same as described in the paralysis assay section. Exactly 50 CL4176 worms were harvested 31 hours after the temperature increase using phosphate-buffered saline (PBS) containing 1% Tween-20 and were washed twice with 1x PBS to remove* E. coli* OP50. Then, the worms were sonicated 4 times for 15 sec each and pipetted into the wells of a 96-well plate containing 200 *μ*l of PBS plus H_2_DCF-DA (final concentration, 50 *μ*M in PBS). The fluorescence was quantified using a Synergy HT microplate fluorescence reader (Bio-Tek Instruments, Winooski, USA) at 485 nm excitation and 530 nm emission. The experiment was independently performed three times.

### 2.6. Quantitative Real-Time RT-PCR (qRT-PCR) Analysis

Worms were collected by washing them with M9 buffer three times and were pelleted by centrifugation at 4,000 rpm for 1 min. The worms were freeze-thawed and transferred directly into 1 mL of TRIzol reagent (Thermo Fisher Scientific, Shanghai, China). After 200 *μ*L of chloroform was added, the worm suspension was shaken vigorously and centrifuged at 12,000 ×g for 10 min. The total nematode RNA in the supernatant was precipitated using isopropanol. The RNA concentration and integrity were measured using a NanoDrop spectrophotometer. The cDNAs were synthesized by reverse transcription using FastQuant RT Kit (with gDNase; Tiangen, Beijing, China) according to the manufacturer's protocol. The expression of the *β*-amyloid gene amy-1 (forward, 5′-CCGACATGACTCAGGATATGAAGT-3′, reverse, 5′-CACCATGAGTCCAATGATTGCA-3′); DAF-16 (forward, 5′- GAAAGAGCTCGTGGTGGGTTATTA-3′, reverse, 5′-TCCGCGGCGAGATTTTTC-3′); sod-3 (forward, 5′-AGCATCATGCCACCTACGTGA-3′, reverse, 5′-CACCACCATTGAATTTCAGCG-3′); and small heat shock protein hsp-16.2 (forward, 5′-CTGCAGAATCTCTCCATCTGAGTC-3′, reverse, 5′-AGATTCGAAGCAACTGCACC-3′) were determined by qRT-PCR performed on a qTOWER 2.2 Real-Time PCR System (Analytik Jena AG, Thuringia, Germany) using SuperReal PreMix Plus (SYBR Green; Tiangen, Beijing, China). The relative levels of gene expression were calculated by the 2^−ΔΔCT^ method using the gene act-4 (forward: 5′-GCCACCGCTGCCTCCTCATC-3′, reverse: 5′-CCGGCAGACTCCATACCCAAGAAG-3′) as the internal control. The experiment was repeated in triplicate.

### 2.7. *C. elegans* RNA Interference (RNAi) Assay

Daf-16 expression was knocked down by feeding the* C. elegans* CL4176 with* E. coli* strain HT115 (DE3) bacteria carrying daf-16 dsRNA. The RNAi plates were prepared with NGM containing 1 mM isopropyl *β*-D-1-thiogalactopyranoside (IPTG) and 50 *μ*g/mL ampicillin.* C. elegans* was fed with* E. coli* HT115 strains expressing dsRNA specific to the daf-16 gene. After 3-4 h, worms were removed and eggs were permitted to mature to L4 young larvae. The L4 larvae were transferred to another plate containing dsRNA and allowed to lay eggs. Subsequently, the resultant adult worms were used for the paralysis assays. Animals fed HT115 bacteria harboring the empty vector, L4440, were used as negative controls.

### 2.8. DAF-16 Nuclear Translocation Quantification

Age-synchronized day 1 adult nematodes of the TJ356 transgenic strain stably expressing a DAF-16::GFP fusion protein as a reporter, pretreated with GNC (3.94 mg/mL) or control, were exposed to 50 *μ*M juglone for 5 min, then immobilized with azide sodium, and placed on 2% agarose pads on a glass slide. The subcellular DAF-16 distribution was analyzed by a Nikon SMZ 1500 fluorescence microscope. Expression patterns of TJ356 worms were classified into three categories (cytosolic, intermediate, and nuclear). At least 20 animals from each group were examined, and the experiment was performed three times independently.

### 2.9. RNA-Seq Analysis of Wild-Type* C. elegans* N2

To further investigate the transcriptional effects of GNC treatment, we analyzed gene expression of* C. elegans* wild-type N2 treated with GNC (3.94 mg/mL) and control at the same age (old worms, 22-days old). Synchronized populations were cultured in S-complete liquid medium containing GNC or a vehicle control (H_2_O), and* E. coli* OP50 were added to the medium as a food source. Total RNA was extracted using RNAiso Plus Total RNA extraction reagent (TaKaRa, China) according to the manufacturer's instructions. RNA was further purified with an RNAClean XP Kit (Beckman Coulter, Inc. Kraemer Boulevard Brea, CA, USA) and an RNase-Free DNase Set (QIAGEN, GmBH, Germany). The samples were sent to Shanghai Biotechnology Corporation for library construction and sequencing using Illumina HiSeq 2500 (Illumina, USA). The sequence quality of the data sets was assessed with an Agilent Bioanalyzer 2100 (Agilent technologies, Santa Clara, CA, USA). Transcript expression levels were estimated using fragments per kilobases per million reads (FPKM) values to allow us to compare different genes or samples. The up- or downregulated genes were identified by filtering the RNA-seq data with the following cut-off: twofold change in the expression level and a false discovery rate (FDR) of less than 0.05. All analyses were performed at Shanghai Biotechnology Corporation (Shanghai, China). Gene expression data determined using next-generation RNA sequencing technology has been deposited in the Gene Expression Omnibus (GEO) database with accession number GSE98195. Publicly available databases, primarily AgriGO (http://bioinfo.cau.edu.cn/agriGO/) for the Gene Ontology (GO) enrichment analysis and KEGG for the pathway enrichment analysis (http://www.genome.jp/kegg/), were used to investigate the biological significance of the differentially expressed genes.

### 2.10. Statistical Analysis

Statistical analyses of differences between groups in the paralysis assays were performed using the log-rank test. Student's *t*-test was performed to compare two data sets. Significant differences between multiple groups were evaluated using one-way analysis of variance (ANOVA) with Duncan's test. GraphPad Prism software 6.0 was used for statistical analyses. Differences were considered significant at a *p* value less than 0.05.

## 3. Results

### 3.1. GNC Delayed the Paralysis of A*β*-Transgenic* C. elegans* CL4176 Nematodes

**Figure 1:**
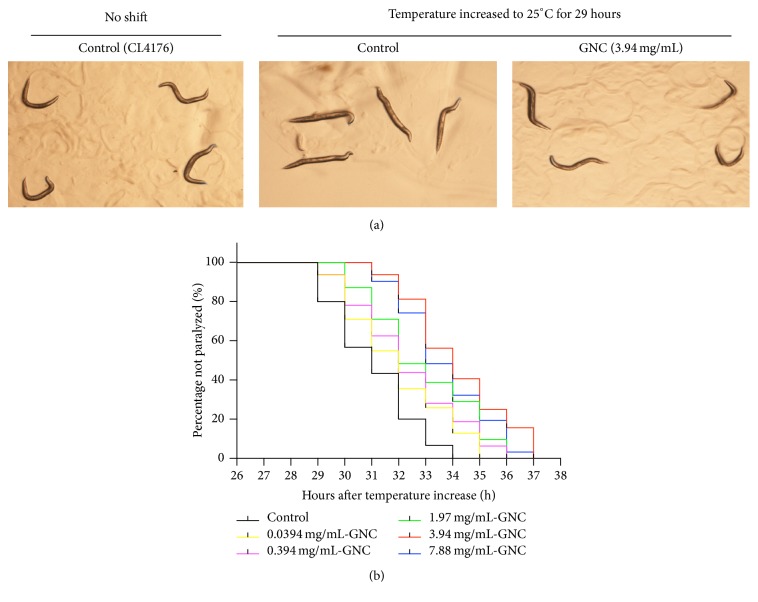
GNC delayed A*β*-induced paralysis in transgenic* C. elegans* CL4176 at 25°C. (a) Representative image of transgenic* C. elegans* CL4176 showing paralysis caused by A*β* expression. Representative images of control worms (no temperature shift) at 16°C and the effect of the temperature increase to 25°C for 29 h to induce A*β* expression in control and GNC-treated (3.94 mg/mL) transgenic* C. elegans* CL4176 are shown. (b) Curves show the course of A*β*-induced paralysis in transgenic* C. elegans* CL4176 treated with a vehicle control (H_2_O) or various doses of GNC. GNC increased the time to paralysis onset in CL4176 worms in a dose-dependent manner (at least 35 worms were tested in each group, and the experiments were performed more than 3 times).

To evaluate whether GNC ameliorated the toxicity of the *β*-amyloid peptide, we used transgenic* C. elegans* CL4176 expressing human A*β*_1–42_ in the muscles. The CL4176 strain displays a phenotype of A*β* production and aggregation when the temperature is increased to 25°C, and progressive paralysis subsequently occurs. We treated CL4176 worms with different concentrations of GNC (0.0394, 0.394, 1.97, 3.94, and 7.88 mg/mL) at 16°C for 48 h and then transferred them to 25°C. As shown in Figure 1, untreated control worms exhibited almost complete paralysis within 34 h at 25°C. In contrast, GNC significantly increased the paralysis time of CL4176 worms. We also found that GNC delayed paralysis in a dose-dependent manner and the maximum increase of paralysis time was observed with 3.94 mg/mL. These observations suggested that GNC may have the potential to protect against *β*-amyloid peptide induced toxicity.

### 3.2. GNC Reduced the Levels of A*β* in* C. elegans* CL4176 by Downregulating the Expression of the A*β* Transgene

**Figure 2:**
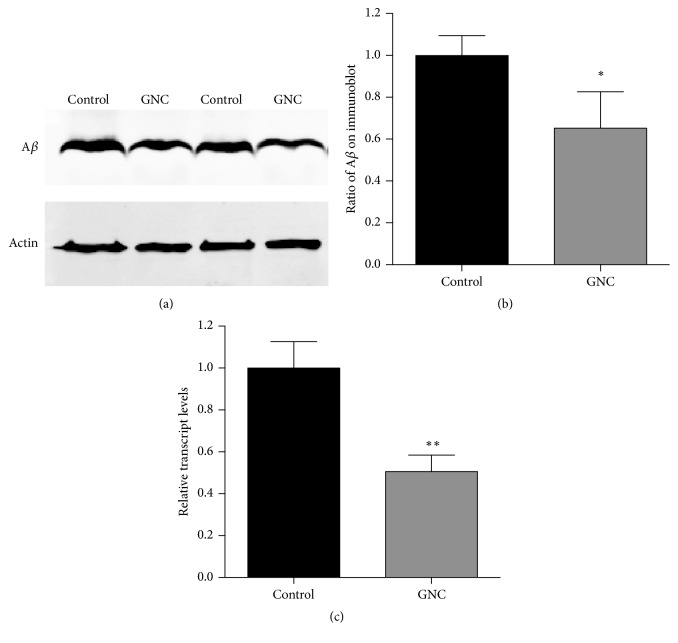
GNC reduced the A*β* mRNA and protein levels in* C. elegans* CL4176. (a) Western blot of the transgenic* C. elegans* CL4176 treated with GNC (3.94 mg/mL) and the control (H_2_O). A representative blot of three independent experiments is shown. (b) The mean optical density was calculated using ImageJ. Quantitative comparisons of the A*β* levels depict a significant 35% reduction in A*β* levels (*p* = 0.0383) in GNC (3.94 mg/mL) pretreated worms. (c) The GNC (3.94 mg/mL) treatment decreased the levels of the amy-1 transcript by 49% (*p* = 0.0044). *β*-Actin (act-4) was used as an endogenous control, and the expression levels were determined by qRT-PCR using the 2^−ΔΔCT^ method. The data are displayed as means ± SD. ^*∗*^*p* ≤ 0.05; ^*∗∗*^*p* ≤ 0.01.

To determine whether GNC delayed onset of paralysis in CL4176 by reducing the A*β* levels, we examined the effect of GNC on the expression of the A*β* transgene and performed Western bolt analysis to monitor A*β* levels. Both GNC-treated and control worms were harvested 31 h after the temperature increase, and parallel populations were processed for qRT-PCR and the Western blotting assay. As shown in Figure 2, treatment with 3.94 mg/mL GNC resulted in a 49% decrease in the expression of the A*β* transgene (*p* = 0.0044), and a significant 35% reduction in A*β* levels (*p* = 0.0383). Thus, GNC potentially delayed the paralysis of* C. elegans* CL4176 by decreasing A*β* transgene expression and A*β* levels, which contribute to the paralysis phenotype of* C. elegans* CL4176.

### 3.3. GNC Reduced Oxidative Stress in* C. elegans* CL4176 Nematodes

**Figure 3:**
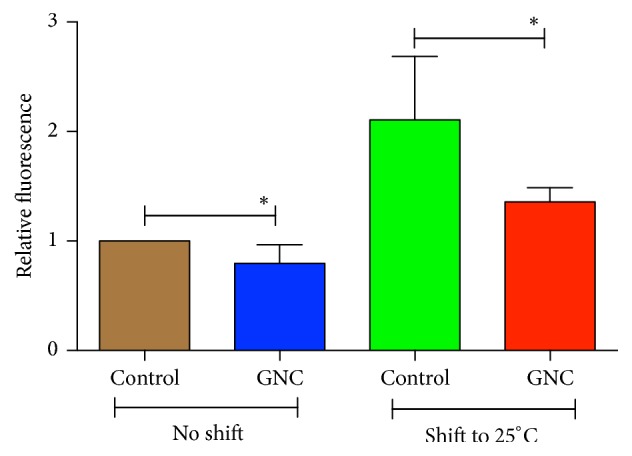
ROS levels in* C. elegans* CL4176 at 16°C or 25°C that had been pretreated with GNC (3.94 mg/mL) or the control. At 16°C (no temperature shift), GNC (3.94 mg/mL) reduced ROS levels by 20% (*p* = 0.033) compared with the vehicle control. When the temperature shifted to 25°C, GNC (3.94 mg/mL) decreased ROS levels by 35.6% (*p* = 0.024) compared to the vehicle control. The data are displayed as means ± SD. ^*∗*^*p* ≤ 0.05.

GNC was previously shown to enhance the resistance of wild-type* C. elegans* N2 to oxidative stress [13]. Because oxidative stress is a major contributing factor to AD, we investigated the ROS levels in the transgenic strain CL4176* in vivo* to determine whether GNC decreased oxidative stress in the AD model worms. At 16°C, GNC (3.94 mg/mL) reduced total ROS levels by 20% (*p* = 0.033) compared with the vehicle control. When the temperature was increased to induce A*β* expression, ROS levels increased in both GNC-treated and control worms (*p* < 0.01). At 31 h after the temperature increase to 25°C, GNC (3.94 mg/mL) also significantly decreased ROS levels by 35.6% (*p* = 0.024) compared to the vehicle control (Figure 3). Based on these results, oxidative stress is associated with the paralysis phenotype caused by A*β* in* C. elegans*, and GNC may protect against A*β* toxicity through its antioxidative stress activity.

### 3.4. Effects of GNC on the Expression of Stress-Induced Genes in* C. elegans*

**Figure 4:**
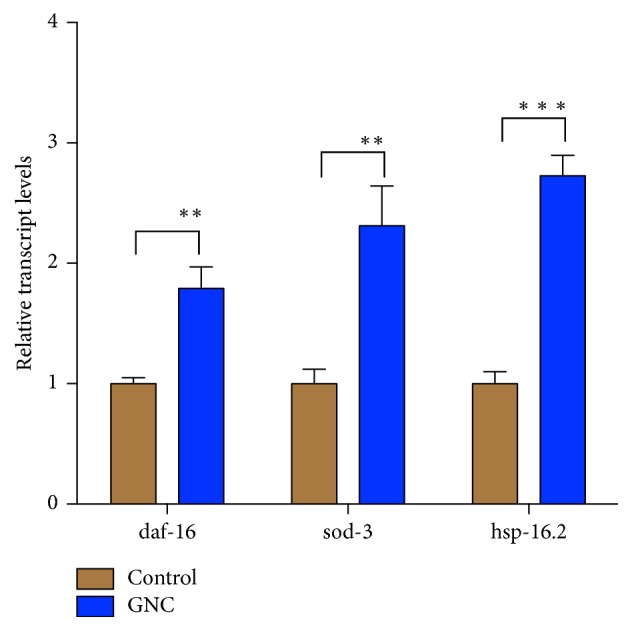
Quantification of mRNA expression levels in GNC-treated worms. The GNC (3.94 mg/mL) treatment affected the relative levels of the daf-16, sod-3, and hsp-16.2 transcripts. *β*-Actin (act-4) was used as an endogenous control, and the expression levels were determined by qRT-PCR using the 2^−ΔΔCT^ method. The data are displayed as means ± SD. ^*∗∗*^*p* ≤ 0.01; ^*∗∗∗*^*p* ≤ 0.001.

To further confirm that GNC increased the resistance of* C. elegans* CL4176 to oxidative stress, we examined the transcript levels of daf-16, superoxide dismutase sod-3, and heat shock protein hsp-16.2 after the temperature increase. Based on the qRT-PCR results, the transcripts of all three genes were expressed at significantly higher levels in worms treated with GNC than in controls at 31 h after the temperature increase (daf-16, 1.8-fold increase, *p* = 0.0017; sod-3, 2.3-fold increase, *p* = 0.0029; hsp-16.2, 2.7-fold increase, *p* = 0.0001) (Figure 4).

### 3.5. DAF-16 Is Required for the Protective Effects of GNC on Paralysis Delay

**Figure 5:**
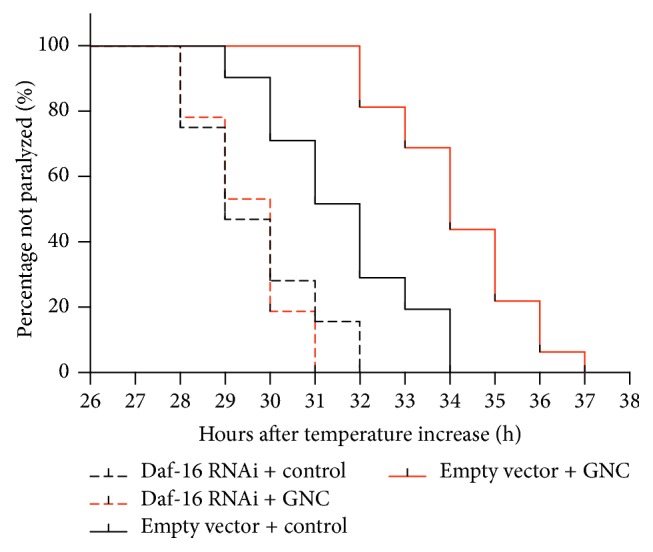
Paralysis in CL4176 with or without Daf-16 knockdown by RNAi. Curves show the course of A*β*-induced paralysis in transgenic* C. elegans* CL4176 (at least 35 worms were tested in each group, and the experiments were performed more than 3 times).

DAF-16 plays an important role in the protection against A*β* toxicity. Our previous study reported that GNC enhances antioxidative stress ability in* C. elegans* N2 via DAF-16 [13]. To further test whether DAF-16 is required for the protective effect of GNC against A*β* toxicity, we performed the experiments by using RNAi knockdown of DAF-16 expression. We found that GNC at 3.94 mg/mL significantly delayed A*β*-induced paralysis in worms grown on bacteria containing empty vector but not on DAF-16 RNAi bacteria (Figure 5). This result indicated that reducing the activity of DAF-16 abolished the protective effect of GNC, suggesting a requirement of DAF-16 for protective effect of GNC.

**Figure 6:**
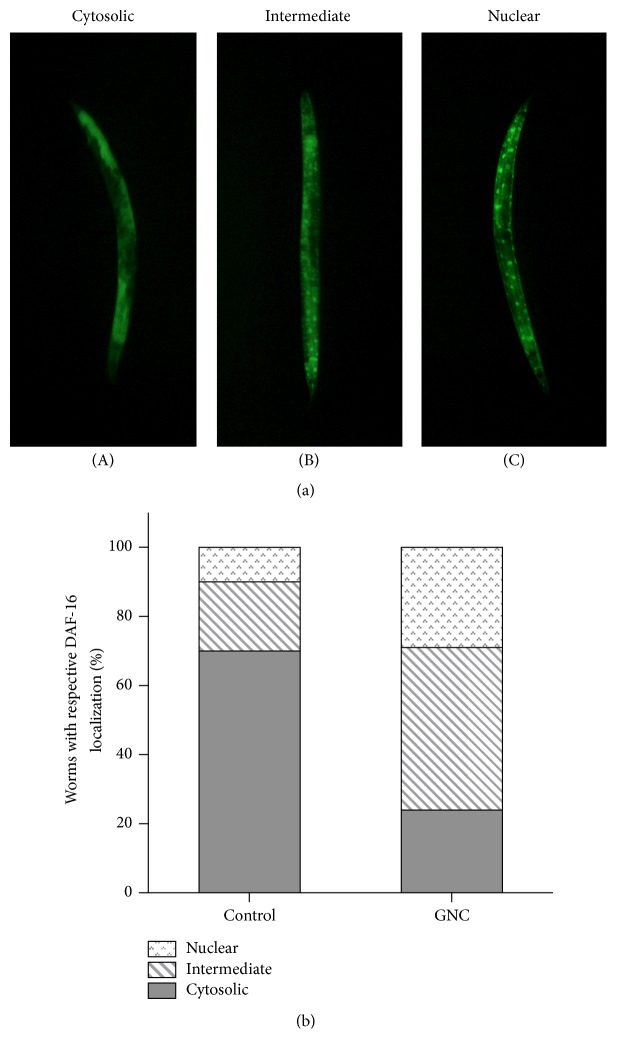
GNC promoted DAF-16 nuclear localization. (a) Representative images for the localization phenotype of DAF-16::GFP in TJ356 adults. Worms were classified into the categories of “cytosolic” (A), “intermediate” (B), and “nuclear” (C) according to their localization phenotypes. (b) Quantization of the distribution of cytosolic, intermediate, and nuclear DAF-16::GFP under oxidative stress conditions. The subcellular distribution of DAF-16 was examined in approximately 20 animals per condition by fluorescence microscopy. The phenotype results were presented in ratio to the whole population at each treatment condition.

To further clarify DAF-16 is required for GNC alleviating A*β* toxicity, we examined the translocation of DAF-16. The subcellular distribution of DAF-16 was classified into the categories of “cytosolic,” “intermediate,” and “nuclear” as representative images showed (Figure 6(a)). Under the juglone-induced oxidative stress conditions, fractions of intermediate and nuclear localization of DAF-16 were greater in GNC-pretreated worms (Figure 6(b)). Therefore, GNC enhanced the translocation of DAF-16 from the cytoplasm to nuclei in oxidative stress.

These findings indicated that DAF-16 is required for the protective effects of GNC on A*β* toxicity.

### 3.6. Genome-Wide Transcriptional Profiling of GNC-Treated* C. elegans* N2

Ageing plays a critical role in AD and remains the most dominant risk factor for AD [32]. The exact pathogenesis of AD is still unclear; it may be connected with ageing, genetic factors, and environmental factors [33]. In addition, AD shares some common signaling pathways with ageing, such as the insulin/IGF-1 signaling pathway, which is the classical and conserved signaling pathway regulated by ageing [34]. Our previous study has showed that GNC extended the lifespan of wild-type* C. elegans* N2 [13]; here, we analyzed the effect of GNC on* C. elegans* N2 gene expression using RNA-seq assays. Differentially expressed genes in response to the GNC treatment were assessed. A total of 1,120 genes were upregulated and 324 genes were downregulated compared to the control treatment. Two different databases, GO Biological Process and KEGG Pathways, were used to investigate the functional classes of differentially expressed genes. Biological processes, including cellular process (GO:0009987), developmental process (GO:0032502), and metabolic process (GO:0008152), were significantly upregulated (*p* < 0.05) in GNC-treated worms (See S. Figure  1 in Supplementary Material available online at https://doi.org/10.1155/2017/7480980). The metabolic pathways affected by GNC were evaluated using KEGG. We identified 5 different KEGG pathways that were associated with the prolongevity effect of GNC (S. Table  1).

## 4. Discussion

To elucidate the regulatory mechanisms of ameliorating *β*-amyloid peptide toxicity by GNC, we performed experiments by using* C. elegans* in this study. We observed that GNC effectively delayed the onset of *β*-amyloid induced paralysis in* C. elegans* CL4176 in a dose-dependent manner. The maximum increase in the time of paralysis onset was delayed by the 3.94 mg/mL treatment. GNC delayed ageing in our previous study, and in this study, we analyzed the sequences of the transcriptomes from GNC-treated and control-treated* C. elegans* using the RNA-seq method. The TGF-beta signaling pathway, Wnt signaling pathway, longevity regulating pathway, FOXO signaling pathway, and oxidative phosphorylation were associated with the lifespan extension effect of GNC. These findings indicated that GNC not only extended the lifespan but also protected against A*β*-induced toxicity in* C. elegans.*

Although the exact cause of AD is still under debate, the amyloid cascade hypothesis is the best accepted aetiology. A*β* deposition leads to an inflammatory response, cytokine release, microglial activation, and reactive astrocytosis; these processes lead to neuronal dysfunction and ultimately large-scale cell death [35]. In this study, GNC suppressed the transcription of amy-1 in* C. elegans* CL4176, suggesting that the delayed onset of paralysis was at least partially due to a reduced amy-1 level. Moreover, GNC significantly reduced A*β* levels compared to controls. Many compounds, such as cocoa peptide [29] and Liuwei Dihuang [28], exert protective effects against A*β* toxicity in the same way. Since A*β* is not endogenously produced in worms, more research is needed to study the effect of GNC on A*β*-induced toxicity.

Oxidative stress is a key factor in the ageing process and has been shown to play a vital role in the pathophysiology of AD [22]. In this study, we measured the ROS levels in* C. elegans* CL4176 and observed a significant increase in ROS levels (more than 2-fold) when the temperature was increased to 25°C, suggesting that ROS production increased in the process of A*β*-induced paralysis. This finding is similar to those observed in AD patients. Sekler et al. observed a close correlation between oxidative stress markers and the stages of AD in patients with AD [36]. According to our previous studies, GNC possesses strong antioxidant activity [13]. We examined whether GNC affected ROS levels in* C. elegans* CL4176 to determine the potential mechanism by which GNC delayed the onset of paralysis. The results showed that GNC reduced total ROS levels under both normal conditions and A*β*-induced toxicity compared with controls. In* C. elegans*, ROS production is a typical outcome of amyloid accumulation, and the effect of GNC on ROS levels may be a consequence of amyloid reduction. However, we cannot exclude the possibility that GNC has direct scavenging effects on ROS, since the antioxidant properties of GNC have also been observed in our previous study [13].

*C. elegans* DAF-16 is the sole ortholog of the FOXO family of transcription factors and plays a central role in promoting cellular antioxidant defenses [37]. DAF-16 activation stimulates the transcription of genes that encode antioxidant enzymes, such as superoxide dismutase (sod). Small heat shock proteins (hsps) are reported to participate in protective responses to the abnormal accumulation of toxic proteins [38]. We evaluated the expression levels of daf-16, sod-3, and hsp-16.2 to further investigate the mechanism by which GNC protects against A*β* toxicity in* C. elegans* and observed a significant increase in the levels of these transcripts in* C. elegans* CL4176 pretreated with GNC. These findings suggest GNC might increase the antioxidative stress ability of* C. elegans* CL4176 via DAF-16. In TCM, many medicinal plants increase the resistance to oxidative stress and have been characterized as potential treatments for mitigating AD. For example, the aqueous extract of* Centella asiatica* reduced oxidative stress in a rat streptozotocin-induced model of AD [39]. As shown in the study by Turgut et al.,* Capparis spinosa* L. exerts a neuroprotective effect by reducing oxidative stress [40]. Our results indicated that the antioxidative stress activity of GNC may be one of the mechanisms by which it protected* C. elegans* from A*β* toxicity.

Next, we further investigated whether GNC requires DAF-16 to protect against A*β* toxicity in* C. elegans* by feeding DAF-16 RNAi bacteria to transgenic CL4176 worms. The results showed that RNAi of DAF-16 significantly eliminated the GNC-mediated decrease of paralysis in AD worms, indicating that the DAF-16 transcription factor is required for GNC protection against A*β* toxicity. Meanwhile, the paralytic phenotype was shortened in DAF-16 knockdown* C. elegans* with or without GNC treatment, which also confirmed that DAF-16 plays an important role in alleviating A*β* toxicity.

To further validate that DAF-16 is required for the protective effect of GNC, we examined the effect of the GNC on the translocation of DAF-16 from the cytoplasm to nuclei. The results showed that GNC enhanced DAF-16 translocation from the cytoplasm to nuclei under oxidative stress conditions. In this study, we found that ROS production increased in the process of A*β*-induced paralysis, suggesting worms were in the oxidative stress conditions. Furthermore, nuclear localization of DAF-16 is a prerequisite for transcriptional activation of its target genes such as sod-3, mtl-1, and clt-2 [41]. In this study, GNC caused a significant increase of sod-3 mRNA levels in CL4176 compared with the untreated worms. Taken together, we speculated that GNC ameliorated A*β* toxicity via DAF-16 pathway.

## 5. Conclusions

In summary, in this study, we reported that GNC, which exhibits prolongevity activity, alleviated A*β*-induced paralysis in* C. elegans* through lowering amy-1 and A*β* levels, reducing ROS, and increasing the daf-16, sod-3, and hsp-16.2 mRNAs, all of which may have mutually exert the protective mechanisms against A*β* toxicity. Furthermore, DAF-16 is essential for the protective effect of GNC. Taken together, our observations suggest that GNC could protect against A*β*-induced toxicity in a* C. elegans* model of AD. Further studies need to be conducted in murine models and humans to analyze the effectiveness of GNC on AD.

## Supplementary Material

S. Figure 1: GO Biological Process analysis of the functional classes of differentially genes. Notes: Biological processes, including cellular process, developmental process and metabolic process were significantly up-regulated (*p* < 0.05) in GNC treated worms. S. Table 1: KEGG pathways related to the prolongevity effect of GNC.
