# Supplementary material for: A Chinese Herbal Formula, Gengnianchun, Ameliorates β-Amyloid Peptide Toxicity in a Caenorhabditis elegans Model of Alzheimer's Disease
Source: Evid Based Complement Alternat Med. 2017 Oct 11;2017:7480980. doi: 10.1155/2017/7480980 (PMC5660795; doi:10.1155/2017/7480980)
Supplement: Supplementary file 1 — S. Figure 1: GO Biological Process analysis of the functional classes of differentially genes. Notes: Biological processes, including cellular process, developmental process and metabolic process were significantly up-regulated (p < 0.05) in GNC treated worms. S. Table 1: KEGG pathways related to the prolongevity effect of GNC. [file 7480980.f1.pdf]

Supplementary material

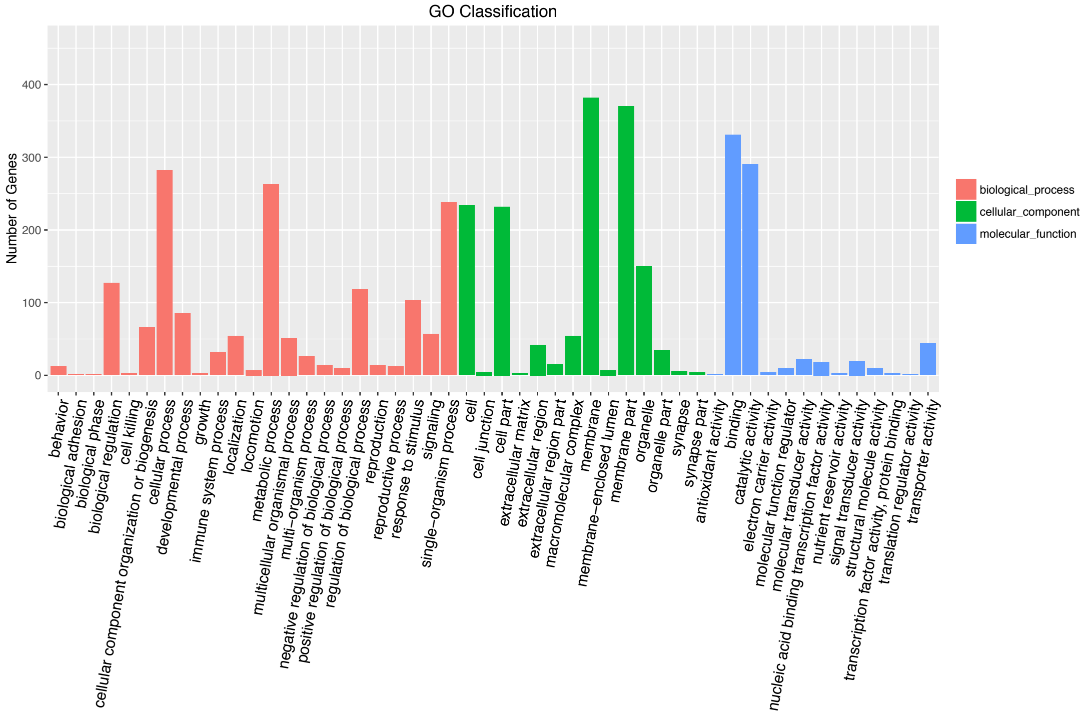

S. Figure 1: GO Biological Process analysis of the functional classes of differentially genes

S. Table 1： KEGG pathways related to the prolongevity effect of GNC

Pathway ID	Pathway description	p-value
cel04350	TGF-beta signalling pathway	9.70E-06
cel04310	Wnt signalling pathway	1.93E-05
cel04212	Longevity regulating pathway	0.149886179
cel04068	FoxO signalling pathway	0.28444136
cel00190	Oxidative phosphorylation	0.467135702
